# Exposure Levels of Airborne Fungi, Bacteria, and Antibiotic Resistance Genes in Cotton Farms during Cotton Harvesting and Evaluations of N95 Respirators against These Bioaerosols

**DOI:** 10.3390/microorganisms11061561

**Published:** 2023-06-12

**Authors:** Atin Adhikari, Pratik Banerjee, Taylor Thornton, Daleniece Higgins Jones, Caleb Adeoye, Sonam Sherpa

**Affiliations:** 1 Department of Biostatistics, Epidemiology & Environmental Health Sciences, Jiann-Ping Hsu College of Public Health, Georgia Southern University, Statesboro, GA 30460, USA; tjthornton65@gmail.com (T.T.); ca13007@georgiasouthern.edu (C.A.); 2 Department of Food Science and Human Nutrition, College of Agricultural, Consumer and Environmental Sciences, University of Illinois at Urbana-Champaign, Urbana, IL 61801, USA; pratik@illinois.edu; 3 Department of Public Health, College of Education, Health, and Human Sciences, The University of Tennessee, Knoxville, TN 37996, USA; dhiggin6@utk.edu; 4 Department of Health Policy and Community Health, Jiann-Ping Hsu College of Public Health, Georgia Southern University, Statesboro, GA 30460, USA; ss35449@georgiasouthern.edu

**Keywords:** bioaerosols, antibiotic resistance genes, respiratory protection, aerobiology, occupational safety, agricultural health, occupational health, fungi, N95 respirator, respiratory protection

## Abstract

The USA is the third-leading cotton-producing country worldwide and cotton farming is common in the state of Georgia. Cotton harvest can be a significant contributor to airborne microbial exposures to farmers and nearby rural communities. The use of respirators or masks is one of the viable options for reducing organic dust and bioaerosol exposures among farmers. Unfortunately, the OSHA Respiratory Protection Standard (29 CFR Part 1910.134) does not apply to agricultural workplaces and the filtration efficiency of N95 respirators was never field-tested against airborne microorganisms and antibiotic resistance genes (ARGs) during cotton harvesting. This study addressed these two information gaps. Airborne culturable microorganisms were sampled using an SAS Super 100 Air Sampler in three cotton farms during cotton harvesting, and colonies were counted and converted to airborne concentrations. Genomic DNA was extracted from air samples using a PowerSoil^®^ DNA Isolation Kit. A series of comparative critical threshold (2^−ΔΔCT^) real-time PCR was used to quantify targeted bacterial (16S rRNA) genes and major ARGs. Two N95 facepiece respirator models (cup-shaped and pleated) were evaluated for their protection against culturable bacteria and fungi, total microbial load in terms of surface ATP levels, and ARGs using a field experimental setup. Overall, culturable microbial exposure levels ranged between 10^3^ and 10^4^ CFU/m^3^ during cotton harvesting, which was lower when compared with bioaerosol loads reported earlier during other types of grain harvesting. The findings suggested that cotton harvesting works can release antibiotic resistance genes in farm air and the highest abundance was observed for phenicol. Field experimental data suggested that tested N95 respirators did not provide desirable >95% protections against culturable microorganisms, the total microbial load, and ARGs during cotton harvesting.

## 1. Introduction

Farmers were believed to be healthier than the people living in urban areas at the beginning of the 20th century because of their exposure to fresh countryside air. This common belief was shown to be incorrect in many previous studies showing that farmers had significantly higher rates of mortality from higher rates of many chronic diseases, such as pulmonary and cardiovascular diseases, on top of their risk of injury [1]. Farmers and agricultural workers are considered to be at an increased risk of asthma [2]. Several previous studies on adult farmers showed higher [3,4,5,6,7] occurrences of asthma than in general populations. A recent US study on respiratory outcomes in 43,548 farmers (NIEHS Agricultural Health Study) showed that participants had a lower prevalence of asthma but a higher prevalence of current respiratory symptoms (wheeze, cough, and phlegm), even after controlling for smoking, body mass index, and population characteristics [8]. Exposure to agricultural dust, which contains fungi, bacteria, microbial toxins and allergens, and hay handling could be some of the important contributors for these increased respiratory symptoms [9,10]. Pulmonary toxins, including microorganisms and organic dusts released during farm works and particle-bound allergic and infectious agents, could be important causal factors for the deteriorated respiratory health of not only farmers but also in nearby rural communities. Respiratory allergy, asthma, and infections are major groups of diseases associated with bioaerosol exposures [11]. Agricultural workers and people living near farms could be at increased risk of occupational respiratory diseases [12]. The role of airborne fungi in the development of respiratory allergies and asthma has been established by previous studies [13,14,15]. More than 80 genera of fungi have been associated with respiratory tract allergy [16,17]. In addition to airborne fungi, high concentrations of bacteria, actinomycetes, and endotoxin were reported in the air of farm workplaces [18,19]. Thermophilic bacteria and spore-forming actinomycetes are well-known sources of allergens [11]. Most bacteria, however, are not very potent allergens, but their cell wall components, such as endotoxin and peptidoglycans, are important pro-inflammatory agents, which may induce respiratory symptoms [11]. Organic dust exposure could be associated with an increased risk of developing non-allergic respiratory disorders, including rhinosinusitis, non-allergic asthma, chronic bronchitis, chronic obstructive pulmonary disease (COPD), and hypersensitivity pneumonitis. Chronic inhalation of complex organic dust rich with microorganisms can increase the severity of all the above-described disorders. Besides human health concerns, the dispersion and deposition of agricultural airborne microorganisms are also important to agricultural researchers because many of them are phytopathogens that typically grow on leaves and stem surfaces or reach there through rainwater or airborne soil dust [20,21]. Exposures to these microbial agents can occur at concentrations significantly higher than in other occupational settings or from ambient sources.

It was reported that harvesting machinery in agricultural farms generates huge amounts of dust [22] and one of our previous studies found a significant increase in the concentration of large dust particles (2–10 µm) during corn harvesting, which was partially attributed to the increase in the concentration of the airborne fungal spores [23]. Several previous studies found or assumed that crop harvesting can significantly increase the concentrations of airborne bacteria [24,25]. A study conducted in Willamette Valley, Oregon, showed that airborne microorganisms in the downwind dust plume of operating grass seed combines can potentially release about 41.9% of the bacteria and 35.1% of the fungi in the airshed in the valley [24]. A study conducted in Iowa performed air sampling on soybean harvesters (combines) and on the farmers in closed cabs as personal samples and reported that personal exposures to microorganisms inside combine cabins ranged from 3.6 × 10^4^ to 4.0 × 10^8^ organisms/m^3^, showing that there are chances of high exposures to organic dust and bioaerosols during soybean harvesting [26]. Another study conducted an aerobiological investigation during cannabis harvesting [27] and found that the cannabis farm workers were potentially exposed to Actinobacteria, as well as the cannabis plant pathogen, namely, *Botrytis cinerea*, during harvesting, bud-stripping, and hand-trimming processes. Except for these few studies, however, there is little published information on the contribution of harvesting activities by machines to the airborne microbial load of the rural areas [28], particularly for cotton harvesting in the state of Georgia, which ranked second in cotton production in the US several years ago, planting >1.4 million acres and farming of cotton is one of the most important factors in Georgia’s agricultural economy [29]. The present study has addressed this information gap.

Because engineering control is not always feasible due to the diverse nature of dust sources during cotton harvesting, the use of dust masks or respirators is one of the most viable options for reducing dust exposure among cotton farmers. However, farmers are often reluctant to use these masks, particularly N95 type of masks or respirators, because they are uncomfortable during hard outdoor farm work and no apparent protective effect was observed because of inappropriate use of the respirators. The OSHA Respiratory Protection Standard (29 CFR Part 1910.134) does not apply to agricultural workplaces. The filtration efficiency of commonly used N95 masks used by farmers was previously tested in laboratory studies (the number 95 in this designation means that the filtration efficiency of these masks is at least 95% at the most penetrating particle size of 0.3 µm) using NaCl particles only. The performances of N95 respirators in cotton harvesting sites can be largely different from the evaluation in laboratory conditions because of the (a) huge loading of dust particles on mask surfaces, which may change the pressure drop and affect penetration; (b) high humidity levels in the state of Georgia; and (c) ambient charged particles settled on surfaces of masks, which can interfere with the filtration efficiency of dust particles. The present study addressed these knowledge gaps as well. Because of the limited budget and resources, we investigated this part by using a manikin-based experimental setup where the real-time filtration efficiency of N95 respirators was examined against particles of different sizes and total microorganisms by simultaneously measuring particle levels inside and outside of the respirator masks fitted on a manikin head form in our existing respiratory evaluation experimental setup. Besides estimating the penetration of total culturable fungi and bacteria, as well as antibiotic resistance genes, we also considered an uncommon ATP measurement method for understanding the total microbial loads inside and outside of the N95 respirators. We considered this method because recently ATP levels as the measures of the total microbial activity or microbial load were explored by several researchers in different kinds of clinical or hospital environmental settings [30,31,32], as well as in field experiments on bioaerosol monitoring and evaluation of bioaerosol samplers [33]. All these combined field experiments provided estimated/simulated workplace protection factors against aerosolized dust and total airborne microorganisms and microbial load during cotton harvesting.

## 2. Materials and Methods

### 2.1. Selection of the Cotton Farms

Three large cotton farms in the Statesboro and Brooklet areas of southern Georgia, United States, were identified for the field experiments. The farmers were contacted by mail and telephone to provide permission to collect environmental samples from their farms during the harvesting season in the fall of 2019. Three harvesting activities occurred in November and one activity occurred in December.

### 2.2. Collection of Air Samples during the Cotton Harvesting Season from the Farm Air and the Interior of the N95 Respirators and the Estimation of the Culturable Microbial Concentrations

**Figure 1:**
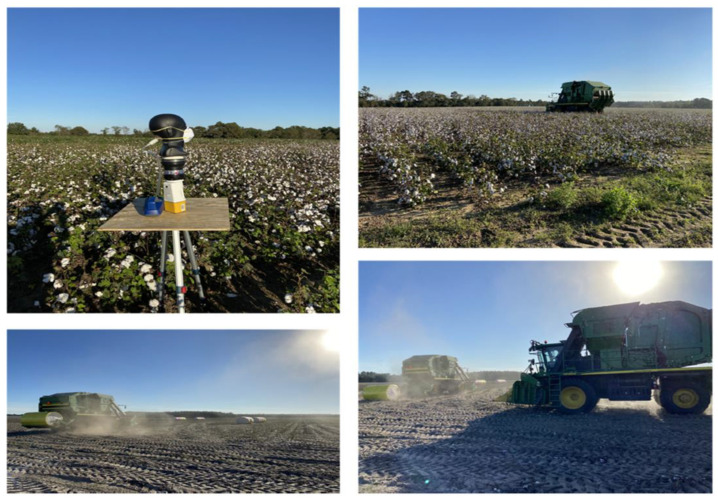
Photographs of cotton farms showing the harvesting work and the air sampling and field N95 respirator evaluation experimental setup.

Air samples were collected during cotton harvesting at each cotton farm using the SAS (Surface Air System) Super 100 Air Sampler (Bioscience Int., Rockville, MD, USA) [Figure 1]. The SAS air sampler was previously used for monitoring airborne microorganisms in various indoor and outdoor occupational and recreational environments [34,35]. In this high-volume sampler, air was aspirated at a 100 L/min flow rate for two to three minutes through an inlet placed at 1.5 m height, which has a series of specially designed small holes. The resulting laminar airflow was directed onto the surface of a nutrient-agar-filled Petri dish. We used a tryptic soy agar (TSA)-containing medium, which is a general-purpose culture medium for the cultivation and isolation of a wide variety of bacteria. Malt extract agar (MEA) medium was used for fungi, including yeasts and molds. After the completion of the sampling cycle, the Petri plates were removed and incubated in the laboratory at 30 ± 2 °C, and colonies of bacteria and fungi were counted after 24–48 h and 72–96 h incubation periods, respectively, and converted to airborne concentrations (CFU/m^3^) after positive hole conversions.

The air samples were collected from inside and outside of the N95 respirators donned on the manikin head by using the SAS sampler, and by using TSA or MEA agar plates. The samples were incubated as described above. The colony counts were converted to interior and exterior culturable fungal or bacterial concentrations (CFU/m^3^).

### 2.3. Analysis of Antibiotic Resistance Genes in the Air Samples

#### 2.3.1. Genomic DNA Extraction

For the molecular analysis of culturable bacteria from the samples collected from three cotton farms, colonies were harvested from TSA plates and the total genomic DNA (gDNA) was extracted using the PowerSoil^®^ DNA Isolation Kit (QIAGEN-MO BIO, Carlsbad, CA, USA), as described previously by our group [36]. 

#### 2.3.2. Polymerase Chain Reaction (PCR) of Antibiotic Resistance Genes

DNA was employed to perform real-time PCR to quantify targeted bacterial major ARGs. The ARGs included β-lactams resistance genes (*bla*_tem−1_ and *bla*_pse−1_) [37], an aminoglycosides resistance gene (*aac*(3)-*Iva*) [37], a tetracycline resistance gene (*tet*A) [37], a trimethoprim resistance gene (*dhfrI*) [37], a sulfomamide resistance gene (*sul*I) [37], a chloramphenicol resistance gene (*flo*) [37], a phenicol resistance gene (*catIII*) [38], a glycopeptide resistance gene (*van*C) [39], and a macrolide resistance gene (*erm*B) [40]. The specifics of the primers are provided in Supplementary Table S1. The positive control for detection used for ARG PCR was *Salmonella typhimurium*. 

A series of comparative critical threshold (ΔΔCT) real-time PCR was used to compare the relative abundance between samples using the formulae ΔC_T_ = C_T,(ARG)_ − C_T,(16S)_ and ΔΔC_T_ = C_T,(Target)_ − C_T,(Ref)_, where C_T_ is the real-time PCR threshold cycle targeting the ARGs with reference to bacterial 16S rRNA gene, as described previously [41]. In general, the PCR reactions were performed using a 20 µL volume containing 6 µL of nuclease-free water (Sigma-Aldrich, St. Louis, MO, USA), 10 µL of SYBR^®^ Green Master Mix (SYBR^®^ Green JumpStart™ Taq ReadyMix, Sigma-Aldrich), 1 µL of forward primer, 1 µL of reverse primer, and 2 µL of DNA in the sample. DNA amplification of the ARGs was carried out in a PCR thermocycler (Bio-Rad CFX96 Real-Time System C1000 Touch Thermocycler, Hercules, CA, USA). The temperature profile for β-lactams, aminoglycosides, tetracycline, trimethoprim, sulfonamides, and chloramphenicol included an initial denaturing step at 95 °C for 10 min; followed by 36 cycles at 95 °C for 30 s, 55 °C for 1 min, and 72 °C for 1 min; and a final step consisting of 72 °C for 7 min [37]. The temperature profile for phenicols included an initial denaturing step at 94 °C for 5 min, followed by 38 cycles at 94 °C for 1 min, 50 °C for 1 min, and 72 °C for 5 min [40]. The temperature profile for glycopeptides included an initial denaturing step at 95 °C for 10 min; followed by 36 cycles at 94 °C for 30 s, 58 °C for 30 s, and 72 °C for 30 s; and a final step consisting of 72 °C for 10 min [38]. The temperature profile for macrolide *erm*B included an initial denaturing step at 94 °C for 5 min; followed by 36 cycles of 94 °C for 1 min, 55 °C for 1 min, and 72 °C for 2 min; and a final extension step at 72 °C for 10 min [39].

### 2.4. Evaluation of N95 Respirators against the Airborne Microorganisms

We assessed the filtration efficiency (simulated protection factor) of two common N95 filtering facepiece respirator models (cup-shaped and pleated, which were sold at nearby stores or online as N95 dust masks) against airborne microorganisms and airborne ARGs in field conditions during cotton harvesting work. We connected the sampling inlet of a SAS Super 100 air sampler with a manikin head with holes drilled at the nostrils. Then the manikin head was donned with the test N95 respirator, exactly in the same way as farm workers wear them in the field. In addition, a few control experiments were conducted where the respirators were fully sealed on the manikin head surfaces. N95 respirators in the United States are certified under NIOSH 42 CFR 84 regulations after passing the tests performed using charge-neutralized sodium chloride aerosol with a particle size of approximately 0.3 µm in diameter [42]. The certification criterion for N95 half-facepiece respirators says that the total momentary particle penetration (P = concentration inside mask/concentration outside mask × 100) through the respirator filter cannot exceed 5% at 85 L/min, i.e., the filtration efficiency, defined as E = 100% − P, must be at least 95%. The advantage of using the SAS air sampler in this experimental setup was its high airflow rate of 100 L/min, which exceeded the NIOSH respirator testing protocol flow rate of at least 85 L/min. We collected air samples from inside and outside of the N95 respirators, one immediately after another, by using this setup and then analyzed the air samples for microorganisms and ARGs as described above.

### 2.5. ATP Measurement of the Interior and Exterior Surfaces of the N95 Respirators

In addition to estimating airborne microorganisms and ARGs, we also measured the ATP levels on exterior and interior surfaces of test N95 respirators as an indicator of the total microbial activity or surface microbial load. We wanted to estimate the surface microbial contaminations of respirators. Furthermore, the inner contamination could be considered an indirect measure of microbial penetration. The ATP bioluminescence assay was used to quantify ATP levels and detect living, metabolically active microbial cells including bacteria and fungal spores or hyphal fragments present in surface dust particles that settled on or penetrated through respirators. ATP levels were measured by swabbing a 10 cm^2^ area on both the inside and outside surfaces of N95 filtering facepiece respirators tested on cotton farms. A rapid and user-friendly commercially available ATP test kit was used for this purpose (UltraSnap™, Hygiena, LLC, Camarillo, CA, USA). According to the manufacturer, this kit uses a unique liquid-stable reagent that provides superior accuracy, longer-lasting signal strength, and more reproducible results. Settled and penetrated dust samples on both surfaces of N95 respirators were collected using sterile cotton swabs and then extracted in the liquid chemical cell releasing reagents and luciferin–luciferase enzyme supplied in test tubes with the test kit for bioluminescence reactions. The bioluminescence from ATP was measured using a portable luminometer, which quantifies the bioluminescent reaction in terms of RLUs (relative light units) from the luminometer digital readout, where the RLU values provide an indirect estimate of the overall microbial activity or microbial load on N95 respirator surfaces.

### 2.6. Statistical Analyses

Descriptive statistics of all data, including means, medians, ranges, and percentages, were calculated using IBM SPSS Statistics 25 software. SigmaPlot 13.0 software was used for creating box plots and calculating the associated statistics.

## 3. Results and Discussion

### 3.1. Concentrations of Airborne Culturable Fungi and Bacteria on the Cotton Farms during Harvesting

**Figure 2:**
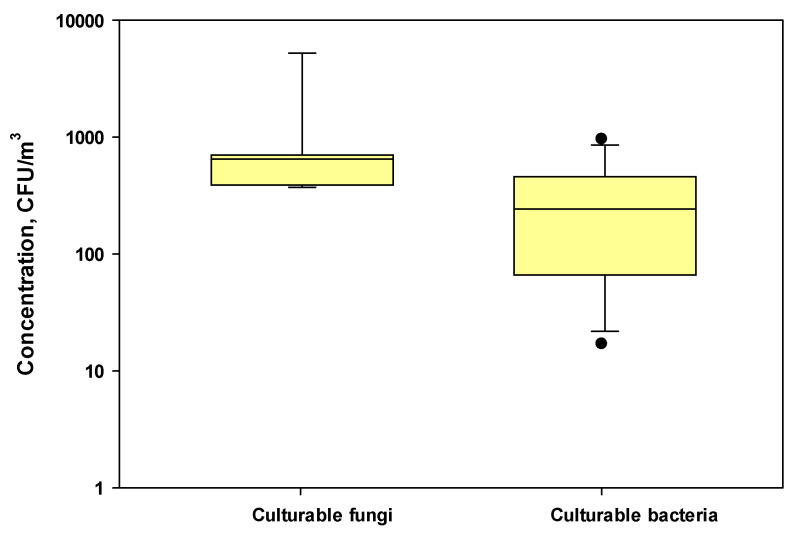
Box plots showing the concentrations of culturable fungi and bacteria in the test farms during cotton harvesting work. The lower and upper boundaries of each box specify the 25th and 75th percentiles, respectively. The line within each box indicates the median and the whiskers above and below each box indicate the 95th and 5th percentiles, respectively.

The mean (±SD) concentrations of culturable bacteria and fungi were 442 ± 309 and 1273 ± 1779 CFU/m^3^, respectively, during harvesting activities at three farms (Figure 2). The concentration ranges were 604–5256 CFU/m^3^ for fungi and 149–963 CFU/m^3^ for bacteria. The concentrations of airborne culturable fungi and bacteria in three nearby control locations without any harvesting activities were 9–192 (mean ± SD = 65.4 ± 50.7) and 17–286 (mean ± SD = 86.7 ± 66.6) CFU/m^3^, respectively.

Typically, the concentrations of airborne fungi and bacteria in agricultural farms during various farming activities ranged from 10^3^ to 10^8^ CFU/m^3^. For example, in our previous study, we found that the concentrations of culturable fungi and bacteria were 8.2 × 10^4^–7.4 × 10^6^ CFU/m^3^ and 0.4 × 10^5^ to 1.4 × 10^6^ CFU/m^3^, respectively, during harvesting on corn farms [23]. The observed concentration levels during cotton harvesting were low, probably due to the low temperature (55–75 °F) during November and December on the days when cotton harvesting work was performed and resulting in the lower growth of microorganisms [43]. A previous study by Lighthart [24] found comparable concentrations of fungi and bacteria in the dust plume from a grass hay-baling operation, which were 2830 and 4410 CFU/m^3^, respectively.

### 3.2. Presence of ARGs in the Collected Air Samples and the Relative Abundance of ARGs with Respect to 16S rRNA Gene Copies

**Figure 3:**
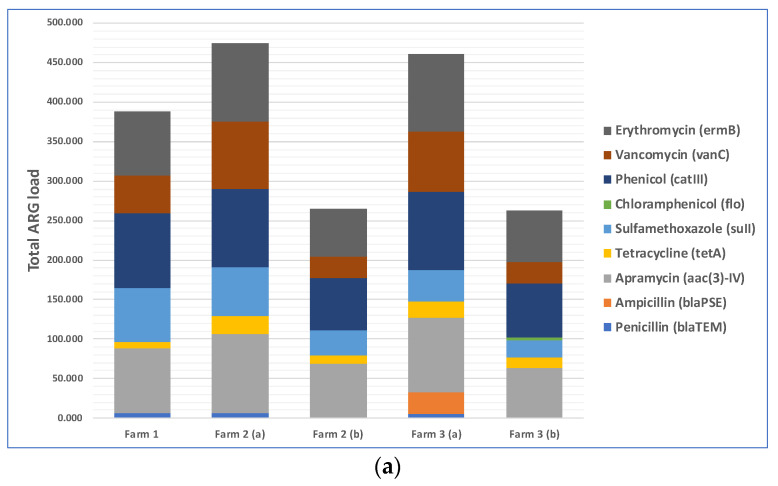

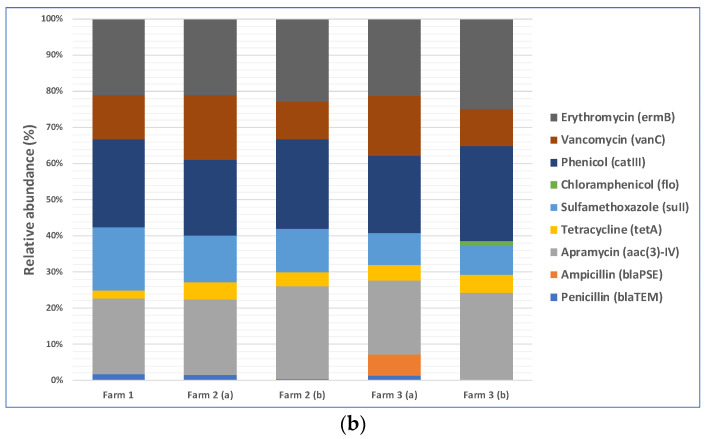
Distribution of nine selected antibiotic resistance genes (ARGs) in the air of three cotton farms during cotton harvesting work: (**a**) total ARG loads in the air samples; (**b**) relative abundance of ARGs in the air samples.

The total loads of ARGs in the air in three farms during cotton harvesting are shown in Figure 3a. Because the total amounts of microorganisms in different air samples were different, the total amounts of antibiotic resistance genes were different too. Therefore, we normalized the concentration of ARGs in air samples to that of the internal reference gene 16S rRNA to obtain the relative abundance of ARGs, which is the percentage equal to the absolute abundance of target genes/the absolute abundance of 16S rRNA genes, following a method described previously [41]. The relative abundances of ARGs in the air in three different farms during cotton harvesting are presented below in Figure 3b. Most of the air samples were positive with respect to the presence of resistance genes for five antibiotics: phenicol, apramycin, erythromycin, vancomycin, and sulfamethoxazole. The spread of phenicol antibiotic resistance genes was previously reported between agriculturally and human-impacted environments and ecosystems [44,45]. Apramycin resistance in bacteria collected from animal farms was reported in previous studies [46,47]. Erythromycin resistance genes in environmental reservoirs, such as farms, hospitals, and watersheds, were reported earlier [48]. As demonstrated by a recent study, animal farms could be potential sources for the exposure risk for airborne transmission of ARGs, where the researchers reported the detection of 18 ARG subtypes (including ARGs such as aadA, cfr, cmlA, fexA, mecA, qnrS, sul1, tetG, and tetW) from airborne microorganisms [49]. Based on this previously published information, it appears that antibiotic resistance in cotton farm bacteria was probably transferred via aerosols from many animal farms located in the rural areas near the farms. These results underscore the need to examine ARG transmission via aerosols, which is a route that was often overlooked in previous studies and may pose a significant exposure risk to farm workers and lead to the spread of ARGs to surrounding areas [50,51]. In addition, irrigation water with antibiotics and ARG residues and the use of compost fertilizer with antibiotic-resistant bacteria in farms may transfer ARGs to farm soils [52,53].

### 3.3. Evaluation of the N95 Respirators against Airborne Culturable Fungi, Bacteria, and ARGs

**Figure 4:**
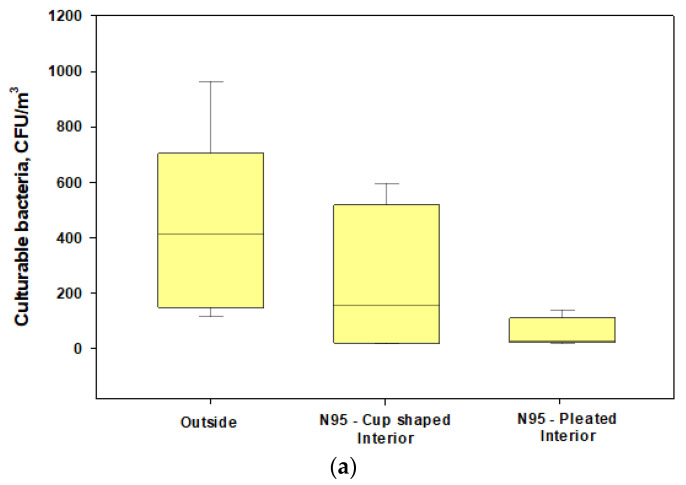

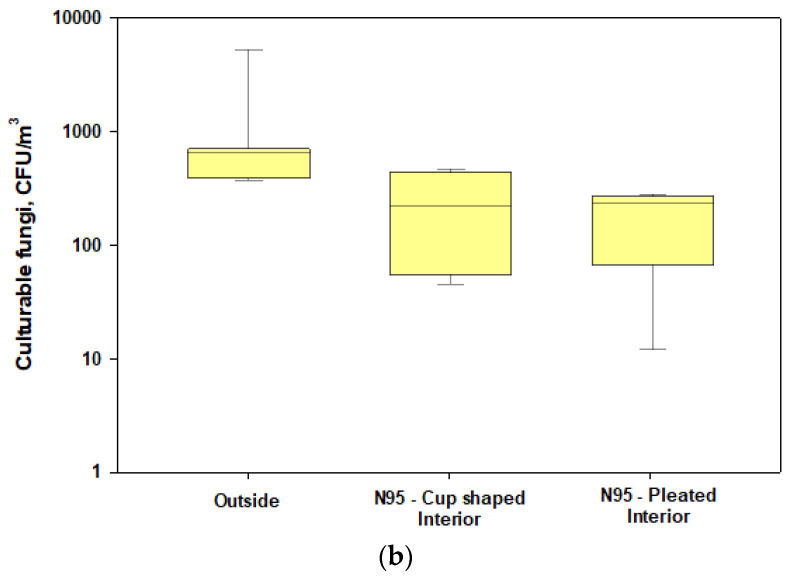
Box plots showing the penetrations of culturable fungi and bacteria through cup-shaped and pleated respirators during harvesting under normal wearing conditions: (**a**) concentrations of culturable bacteria in the interior and exterior spaces of the respirators; (**b**) concentrations of culturable fungi in the interior and exterior spaces of the respirators. The lower and upper boundaries of each box specify the 25th and 75th percentiles, respectively. The line within each box indicates the median and the whiskers above and below each box indicate the 95th and 5th percentiles, respectively.

**Figure 5:**
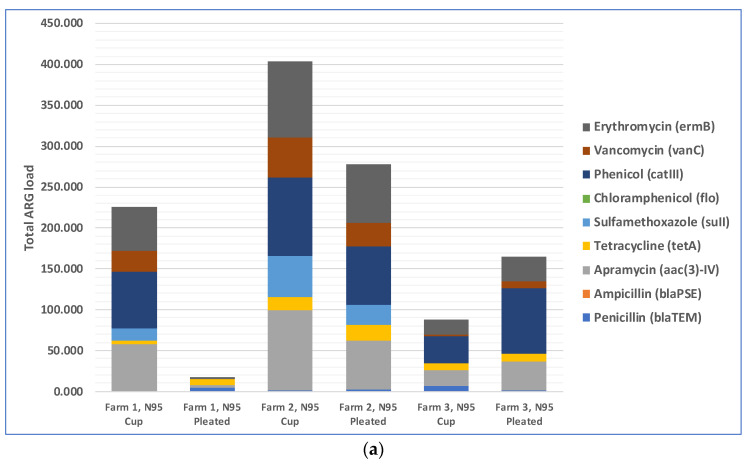

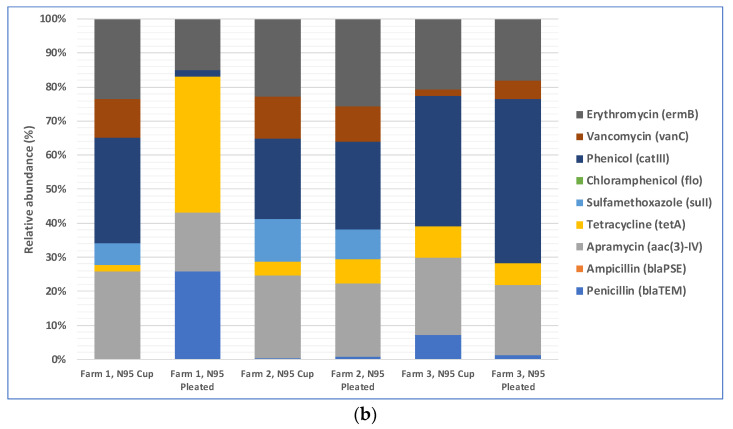
Penetration of ARGs through N95 respirators: (**a**) total ARGs load in the air samples collected from inside of the respirators; (**b**) relative abundance of ARGs in the air samples collected from interiors of the respirators.

We found that penetrations of culturable bacteria exceeded 5% in all experiments for both the cup-shaped and pleated models of N95 respirators (Figure 4a). Similar findings were observed for culturable fungi except for two cases (Figure 4b). The pleated model provided better protection for fungi. Penetration levels of ARGs were >5% in most cases when compared with the total ARGs loads in the exteriors of the respirators (Figure 3a) and the highest abundance and penetration was observed for phenicol (Figure 5a,b). These observations show that tested N95 respirators did not provide the desirable >95% protection against culturable microorganisms, total microbial load, and ARGs during cotton harvesting.

### 3.4. Microbial Loads on the Interior and Exterior Surfaces of the Tested N95 Respirators

**Figure 6:**
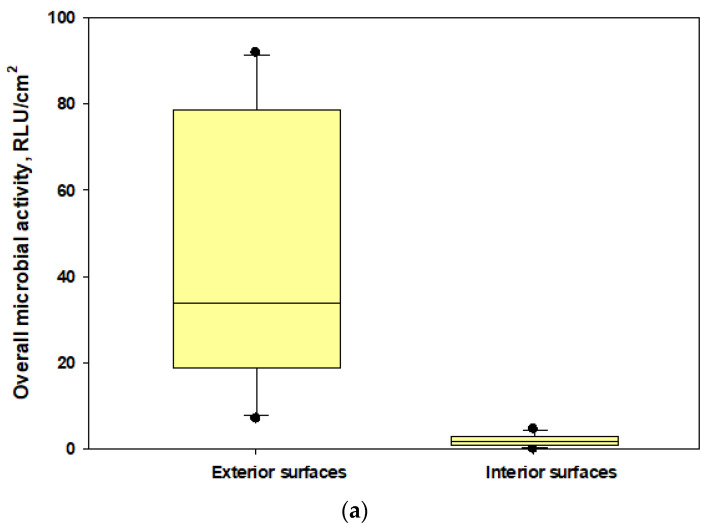

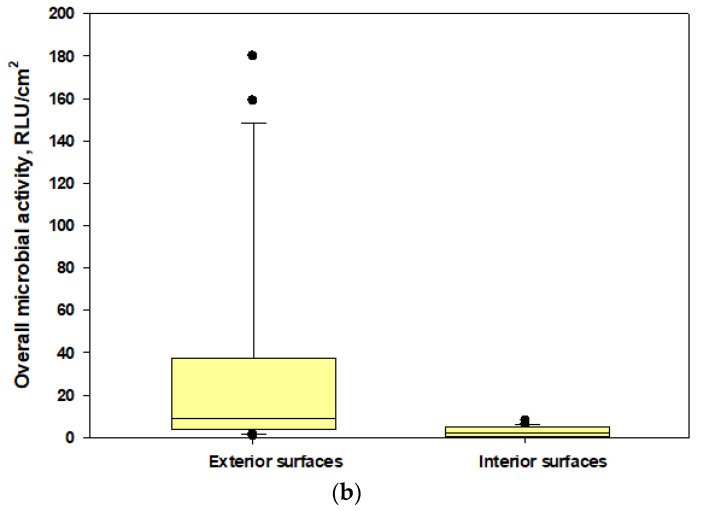
The box plots showing overall microbial loads on the interior and exterior surfaces of tested N95 respirators: (**a**) N95 cup-shaped respirators; (**b**) N95 pleated respirators. The lower and upper boundaries of each box specify the 25th and 75th percentiles, respectively. The line within each box indicates the median and the whiskers above and below each box indicate the 95th and 5th percentiles, respectively.

The data showed that microbial loads on the interior surfaces of the two unsealed N95 masks (mean ± SD: 1.7 ± 1.38 and 2.56 ± 2.48 RLU/cm^2^, respectively) were 3.7–8% of the microbial loads measured in exterior surfaces (mean ± SD: 45.9 ± 31.78 and 31.79 ± 49.77 RLU/cm^2^, respectively) [Figure 6]. The interior surface of the sealed N95 cup-shaped respirator (control) had RLU levels below the lower limit of detections when compared with the exterior surface (125.0 ± 136.74 RLU/cm^2^). This part of our study revealed that metabolically active microorganisms could be present on the interior surfaces of respirators at lower levels compared with the exterior surfaces; however, when we calculated the differences, we found that the 95% protection level may not be achievable (as the interior microbial load percentages ranged up to 8% of the exterior loads) for active microbial load exposures if respirators are not properly worn and face seal leaks occur during active conditions of cotton harvesting work. 

These findings are important because farmers engaged in work with heavy dust exposure risks in outdoor farms typically use N95 filtering facepiece respirators or masks, and often they use the same mask repeatedly. To our knowledge, the growth or activity of microorganisms on filters of respirators upon use in agricultural farms was never tested. The findings from these experiments provide important information on the suitability of respirators or masks for repeated use so that the respirator itself cannot be the source of microbial infections for the user. Second, information on microbial loads on the exterior and interior surfaces of respirators used on farms can also provide important indirect information about the filtration efficiency of respirators against metabolically active microorganisms and microbial fragments.

This field study had several limitations. First, due to the limited number of consenting farmers, the study was conducted on only a few cotton farms. Second, the evaluation of respirators was conducted using a manikin-based setup, which is not exactly the NIOSH-recommended setup, and we could not calculate the workplace protection factors offered by the test N95 respirators because farmers were not using the test respirators during the harvesting tasks. Third, many airborne bacteria and fungi may not be culturable in the selected general-purpose nutrient agar media (TSA and MEA). Finally, we could not analyze the diversity and abundance of fungal isolates in this study because of limited resources.

## 4. Conclusions

Overall, we found that the culturable microbial exposure levels ranged between 10^3^ and 10^4^ CFU/m^3^ during cotton harvesting, which was lower when compared with bioaerosol loads reported during other types of grain harvesting. The analysis of ARGs in air samples clearly suggests that cotton harvesting works can release antibiotic resistance genes in farm air and the highest abundance was observed for phenicol. The manikin-based field experimental data suggest that tested N95 respirators did not provide desirable >95% protection against culturable microorganisms, total microbial load, and ARGs during cotton harvesting. Therefore, additional experiments should be conducted to determine the workplace protection factors offered by these respirators against bioaerosols and ARGs in agricultural farms.

## Data Availability

All data collected are presented and included in the article.

## Supplementary Materials

The following supporting information can be downloaded at: https://www.mdpi.com/article/10.3390/microorganisms11061561/s1, Table S1: Primer sets used in this study and their target ARGs and antibiotic classification. References [41,54] are cited in the Supplementary Materials.
