# Supplementary material for: Exposure Levels of Airborne Fungi, Bacteria, and Antibiotic Resistance Genes in Cotton Farms during Cotton Harvesting and Evaluations of N95 Respirators against These Bioaerosols
Source: Microorganisms. 2023 Jun 12;11(6):1561. doi: 10.3390/microorganisms11061561 (PMC10302439; doi:10.3390/microorganisms11061561)
Supplement: Supplementary file 1 [file microorganisms-11-01561-s001.zip › microorganisms-2428526-supplementary.pdf]

Table S1. Primer sets used in this study and their target ARGs and antibiotic classification

Antibiotics	ARG	Forward primer (5′-3′)	Reverse primer (5′-3′)	Reference
Aminoglycoside	*aac(3)-IVa*	CGTCGCCGAGCAACTTG	CGGTACCTTGCCTCTCAAACC	[54]
β-Lactams	bla_{TEM-1}	TTGACGCCGGGCAAGA	TGCTTTTCTGTGACTGGTGAGTACT	[54]
	bla_{PSE-1}	TTGTGACCTATTCCCCTGTAATAGAA	TGCGAAGCACGCATCATC	[41]
Chloramphenicol	*flo*	ATTGTCTTCACGGTGTCCGTTA	CCGCGATGTCGTCGAACT	[41]
Glycopeptide	*van*C	ACAGGGATTGGCTATGAACCAT	TGACTGGCGATGATTTGACTATG	[41]
Macrolide	*erm*(B)	TAAAGGGCATTTAACGACGAAACT	TTTATACCTCTGTTTGTTAGGGAATTGAA	[41]
Phenicol	catIII	GCACTCGATGCCTTCCAAAA	AGAGCCGATCCAAACGTCAT	[41]
Sulfonamide	*sulI*	CAGCGCTATGCGCTCAAG	ATCCCGCTGCGCTGAGT	[41]
Tetracycline	*tetA*	CTCACCAGCCTGACCTCGAT	CACGTTGTTATAGAAGCCGCATAG	[54]
Trimethoprim	*dhfrI*	GGAATGGCCCTGATATTCCA	AGTCTTGCGTCCAACCAACAG	[41]
Normalization				
16S rRNA	-	*GGGTTGCGCTCGTTGC*	*ATGGYTGTCGTCAGCTCGTG*	[41]
